# Comparative Expression Analysis of Rice and *Arabidopsis* Peroxiredoxin Genes Suggests Conserved or Diversified Roles Between the Two Species and Leads to the Identification of Tandemly Duplicated Rice Peroxiredoxin Genes Differentially Expressed in Seeds

**DOI:** 10.1186/s12284-017-0170-5

**Published:** 2017-06-24

**Authors:** Yun-Shil Gho, Sun-A Park, Sung-Ruyl Kim, Anil Kumar Nalini Chandran, Gynheung An, Ki-Hong Jung

**Affiliations:** 1 Graduate School of Biotechnology & Crop Biotech Institute, Kyung Hee University, Yongin, 17104 Republic of Korea; 2 Plant Breeding, Genetics, and Biotechnology Division, International Rice Research Institute, Metro Manila, Philippines

**Keywords:** Arabidopsis, *cis*-acting regulatory elements, Rice, Peroxiredoxin family, Gus, Tandem duplication

## Abstract

**Background:**

Peroxiredoxins (PRXs) have recently been identified as plant antioxidants. Completion of various genome sequencing projects has provided genome-wide information about PRX genes in major plant species. Two of these -- *Oryza sativa* (rice) and *Arabidopsis* -- each have 10 PRX members. Although significant progress has been made in understanding their biological roles in *Arabidopsis*, those functions in rice, a model crop plant, have not been well studied.

**Results:**

We performed a comparative expression analysis of rice and *Arabidopsis* PRXs. Our phylogenetic analysis revealed that one subgroup contains three rice and three *Arabidopsis* Type-II PRXs that are expressed ubiquitously. This suggests that they are involved in housekeeping functions to process reactive oxygen species (ROS). Within the second subgroup, expression of *Os1-CysPrxA (LOC_Os7g44430)* and *AtOs1-CysPrx* is conserved in seeds while *Os1-CysPrxB (LOC_Os7g44440)* shows a root-preferential pattern of expression. We used transgenic plants expressing the GUS reporter gene under the control of the promoters of these two tandem duplicates to confirm their meta-expression patterns. Our GUS expression data from developing seeds and those that were germinating indicated that *Os1-CysPrxB* is involved in root development, as initiated from the embryo, while *Os1-CysPrxA* has roles in regulating endosperm development near the aleurone layer. For the third and fourth subgroups, the rice PRXs are more likely to show leaf/shoot-preferential expression, while those from *Arabidopsis* are significantly expressed in the flowers and seeds in addition to the leaf/shoot. To determine the biological meaning of those expression patterns that were dominantly identified in rice PRXs, we analyzed three rice genes showing leaf/shoot-preferential expression in a mutant of the light-responsive *1-deoxy-D-xylulose 5-phosphate reductoisomerase* (*dxr*) gene and found that two of them were significantly down-regulated in the mutant.

**Conclusion:**

A global expression analysis of the PRX family in rice identified tandem duplicates, *Os1-CysPrxA* and *Os1-CysPrxB,* in the 1-CysPrx subgroup that are differentially expressed in developing seeds and germinating seeds. Analysis of the *cis*-acting regulatory elements (CREs) revealed unique CREs responsible for embryo and root or endosperm-preferential expression. In addition, the presence of leaf/shoot-preferential PRXs in rice suggests that they are required in that crop because those plants must tolerate a higher light intensity in their normal growth environment when compared with that of *Arabidopsis*. Downregulation of two *PRX*s in the *dxr* mutant causing an albino phenotype, implying that those genes have roles in processing ROS produced during photosynthesis. Network analysis of four PRXs allowed us to model regulatory pathways that explain the underlying protein interaction network. This will be a useful hypothetical model for further study.

**Electronic supplementary material:**

The online version of this article (doi:10.1186/s12284-017-0170-5) contains supplementary material, which is available to authorized users.

## Background

Peroxiredoxins (PRXs) catalyze the decomposition of peroxides and function in diverse compartments to protect cells against damage from reactive oxygen species (ROS) (Dietz 2003). The PRX family contains thiol-dependent peroxidases that are evolutionarily widespread in bacteria, fungi, animals, cyanobacteria, and plants (Umate 2010; Dietz et al. 2006; Bhatt and Tripathi 2011). Completion of several genome sequencing projects has provided genome-wide information about PRX genes in major plant species. According to their primary structure, they have been classified into four functional subgroups: PrxQ, 1-CysPrx, 2-CysPrx, and Type-II Prx (Umate 2010). In *Suaeda salsa*, PrxQ helps determine tolerance to abiotic stresses such as cold and salt (Jing et al. 2006), 1-CysPrx is involved in delaying seed germination under abiotic stress (Haslekas et al. 2003), PrxIIE represses protein nitration and is active in pathogen defenses (Romero-Puertas et al. 2007), while PrxIIF regulates root growth in the presence of cadmium and salicylhydroxamic acid (Haslekas et al. 2003). Two model systems, *Oryza sativa* (rice) and *Arabidopsis thaliana* (herein *Arabidopsis*), have 10 and 11 PRX members, respectively, in their genomes (Umate 2010). Based on their subcellular localization, the PRXs in *Arabidopsis* comprise four subgroups: four PRXs (2-CysPrxA, 2-CysPrxB, PrxQ, and PrxIIE), localized in the chloroplast; a PrxIIF, in the mitochondrion; a 1-CysPrx, in the nucleo-cytoplasm; and three (PrxIIB, PrxIIC, and PrxIID), in the cytosol (Dietz 2011). In *Arabidopsis*, 2-CysPrx is involved in growth and photosynthesis (Baier and Dietz 1999; Pulido et al. 2010). For rice, however, no genetic studies have revealed functional roles for any PRXs except rice 1 Cys-peroxiredoxin (R1C-Prx/Os1-CysPrxA), which acts as a dormancy regulator and an antioxidant, based on a study with transgenic plants of tobacco, an heterologous system, that constitutively express *R1C-Prx* (GenBank Accession Number C19186; Lee et al. 2000).

Expression profiles under diverse developmental stages and growth conditions are a simple and powerful tool for obtaining information about genes for which functions have not been characterized (Chandran et al. 2016a, 2016b). We recently suggested putative functions for most members of several gene families based on the integration of experimental and meta-expression data (Jung et al. 2013; Jin et al. 2012; Nguyen et al. 2013; Nguyen et al. 2014; Nguyen et al. 2015). Applying a similar approach with the rice PRX family could help elucidate the functions of those members.

The 2-C methyl-D-erythritol 4-phosphate (MEP) pathway is a unique and essential process for bacteria, algae, and plants (Proteau 2004). The final product, isopentenyl pyrophosphate, is used for the synthesis of diverse secondary metabolites such as isoprenoids, carotenoids, chlorophylls, and tocopherols, as well as for hormones such as gibberellins and abscisic acid (Jung et al. 2008). We recently identified a T-DNA insertional mutant in a gene encoding the second enzyme in this pathway, 1-deoxy-d-xylulose 5-phosphate reductoisomerase (DXR; EC 1.1.1.267). Under greenhouse conditions, this mutant exhibits an albino phenotype at the early seedling stage (Jung et al. 2008). Co-expression of genes in the MEP pathways with those in the pathways for chlorophyll and carotenoid biosynthesis implies a potential relationship among those pathways during photosynthesis (Jung et al. 2008). In addition, the photosystems are major sources for generating ROS during photosynthesis (Pospisil 2009). Because the four PRXs in rice are localized to the chloroplast (Dietz et al. 2006), we are very interested in learning how they are involved in light responses or photosynthesis.

Here, we performed a comparative expression analysis, within the context of a phylogenic tree, using PRX family genes from rice and *Arabidopsis*. Our results suggested probable functional orthologs between specific pairs. Tandemly duplicated *Os1-CysPrxA/LOC_Os07g44430* and *Os1-CysPrxB/LOC_Os07g44440* showed expression that was distinctly preferential in both developing seeds and germinating seeds. We then used transgenic plants expressing the GUS reporter gene under the control of their promoters to confirm the pattern of differential expression by these two tandem duplicates. Finally, we identified unique *cis*-acting regulatory elements (CREs) for root-, embryo-, or endosperm-preferential expression in rice. In the rice mutant *1-deoxy-D-xylulose 5-phosphate reductoisomerase* (*dxr*), which is defective in the light response, downregulation of two *PRX* genes indicated the involvement of those proteins in *DXR*-mediated light signaling.

## Results

### Identification of rice peroxiredoxin genes and comparative phylogenic analysis with *Arabidopsis* PRX genes

**Fig. 1:**
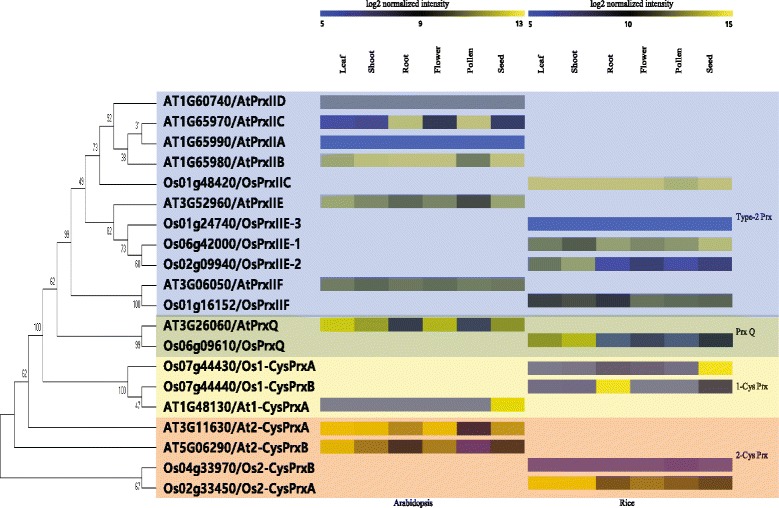
Comparative meta-expression analysis of rice and *Arabidopsis* PRX family genes in six tissues/organs. Data from leaf, shoot, root, flower, pollen, and seed samples of both species were combined to produce phylogenetic tree. *Yellow*, *high level* of expression; *blue*, *low level*. Numeric values indicate average of normalized log_2_ intensity of microarray data. Names of subgroups are on *right side* of heatmap. Heatmaps in *middle* indicate meta-expression data for *Arabidopsis* PRX genes on *left* and rice PRX genes on *right*

Umate (2010) recently performed a phylogenetic analysis of 11 PRX family members of rice and 10 in *Arabidopsis*. However, unlike for the latter, roles for the individual members in rice have not been well characterized. Therefore, our objective was to assign biological functions to rice PRX genes according to developmental stage or type of tissue/organ. Our phylogenic analysis with genes from each species indicated that one rice member (LOC_Os07g15670) was not clustered with the others (data not shown). After removing that one, we had 10 from each species to examine. In all, four subgroups were revealed: 1-CysPrx, 2-CysPrx, PrxQ, and Type-II Prx (Fig. 1). Five rice and six *Arabidopsis* PRXs were assigned to the Type-II Prx subgroup, two rice and one *Arabidopsis* to the 1-CysPrx subgroup, two rice and two *Arabidopsis* to the 2-CysPrx subgroup, and one each for rice and *Arabidopsis* to the PrxQ subgroup. We hypothesized that the PRXs of both species that clustered within the same subgroup could have similar biological functions. The tandem duplicates included three *Arabidopsis PRX*s (*AtPrxIIA*, *AtPrxIIB*, and *AtPrxIIC*) on Chromosome 1 and two rice *PRX*s (*Os1-CysPrxA* and *Os1-CysPrxB*). We believe that these duplicates are probably redundant. All of the remaining genes were independently located on different chromosomes (Additional file 1: Figure S1).

### Functional assignment of rice and *Arabidopsis* PRX genes using meta-expression analysis of six tissues/organs

Although the results from our phylogenetic analysis allowed us to infer functional similarity among rice and *Arabidopsis* PRX genes clustered within the same subgroup, we performed additional experiments to determine whether those functions were conserved between species. Expression patterns for most of these *PRX*s were investigated based on a large collection of microarray data (Fig. 1 and Additional file 2: Figure S2). The exception was *AtPrxIID*, for which no probes were available on the Affymetrix array we used.

Within the Type-II Prx subgroup, OsPrxIIC clustered together with AtPrxIIA, AtPrxIIB, AtPrxIIC, and AtPrxIID. Because its expression pattern was the most similar to that of *AtPrxIIB*, we inferred that they are functional orthologs. We found it interesting that, although *AtPrxIIA*, *AtPrxIIB,* and *AtPrxIIC* are tandemly duplicated, their expression patterns were not similar. Unlike *AtPrxIIB*, which was ubiquitously expressed, *AtPrxIIC* showed root- and pollen-preferential expression, suggesting its possible role in the development of those organs in *Arabidopsis*. Expression of *AtPrxIIA* was suppressed overall, suggesting a loss-of-function during evolution or possible suppression through epigenetic regulation. Although OsPrxIIC, OsPrxIIE2, and OsPrxIIE3 clustered with AtPrxIIE, none of the first three showed a pattern of expression similar to that of *AtPrxIIE*. OsPrxIIF clustered with ATPRXIIF; both of these genes were ubiquitously expressed but differed in their patterns. In the Type-II Prx subgroup, we identified six *Arabidopsis* and five rice members. We concluded that those genes with ubiquitous expression patterns could have similar functions. However, the root- and pollen-preferential functioning of AtPrxIIC versus leaf- and shoot-preferential functioning of OsPrxIIE2 suggested that their expression is specific to one species only.

In the 1-Cys Prx subgroup, Os1-CysPrxA and Os2-CysPrxB clustered with At1-CysPrx. Genes in this cluster showed tissue- or organ-specific expression patterns. *Os1-CysPrxB* was root-preferential while *Os2-CysPrxA* and *At1-CysPrx* were preferentially expressed in the seed. This indicated that the seed-related functions of genes in this subgroup might be conserved between species. We found it interesting that expression of *Os1-CysPrxB* was root-preferential in rice, suggesting a functional role in root development in this crop. The promoters of *Os1-CysPrxA* or *Os1-CysPrxB* likely contain CREs that determine root- or seed-preferential expression. In the *PrxQ* subgroup, *OsPrxQ* and *AtPrxQ* presented similar patterns in various tissues/organs, but expression of the former was much higher in the flowers and seeds when compared with the latter. In the *2-Cys Prx* subgroup, expression in the pollen and seed was somewhat similar among *Os2-CysPrxA*, *At2-CysPrxA*, and *At2-CysPrxB*. Although *Os2-CysPrxB* was expressed at a very low level overall, it was most highly expressed in pollen, indicating that genes in that subgroup have unique functions in the development of rice pollen and *Arabidopsis* seed.

AtPrxIIC showed root- and pollen-preferential expression patterns. Although it is a possible functional ortholog of *Os1-CysPrxB* in root development, these two genes did not show a close evolutionary relationship. Leaf/shoot-preferential expression was only identified in the rice PRX family (three members), while five *Arabidopsis PRX*s and two rice *PRX*s were highly expressed in the seed.

### Validation of meta-expression patterns of rice PRX genes using RT-PCR analysis

**Fig. 2:**
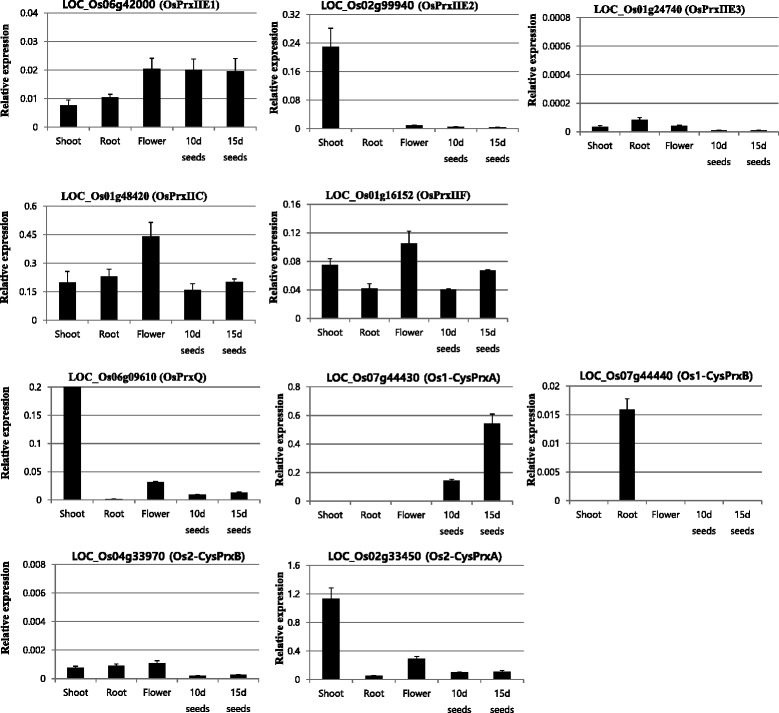
Validation of meta-expression patterns in six tissues/organs for 10 PRX genes in rice based on qRT-PCR analysis of samples from seedling shoots, roots, flowers, seeds at 10 DAP, and seeds at 15 DAP. *Rice ubiquitin 5 (OsUbi5, LOC_Os01g22490*) was used as internal control. Y-axis, expression level relative to *OsUbi5*; X-axis, samples used for analyses

To confirm the meta-expression patterns we had observed for the rice PRX genes, we conducted RT-PCR analysis with seedling shoots, roots, flowers, and developing seeds at 10 or 15 days after pollination (DAP). For the five genes in the type-II Prx subgroup, three (*OsPrxIIC*, *OsPrxII E1*, and *OsPrxIIF*) were ubiquitously expressed in the tested tissues/organs (Fig. 2). *OsPrxIIE2* showed a shoot-preferential expression pattern but expression of *OsPrxIIE3* was very low overall. *OsPrxQ* was expressed at the highest level in the shoots (Fig. 2). The latter gene was also significantly expressed in flowers. *Os1-CysPrxA* showed the highest expression in developing seeds while *Os1-CysPrxB* in the *1-Cys Prx* subgroup was highly expressed in the roots. Although expression of *Os2-CysPrxA* was highest in the shoots, that of *Os2-CysPrxB* was very low overall. These data were consistent with our meta-expression data, indicating that the latter, when based upon a large collection of reference data, are reliable.

### GUS expression analysis of two tandemly duplicated rice PRX genes

**Fig. 3:**
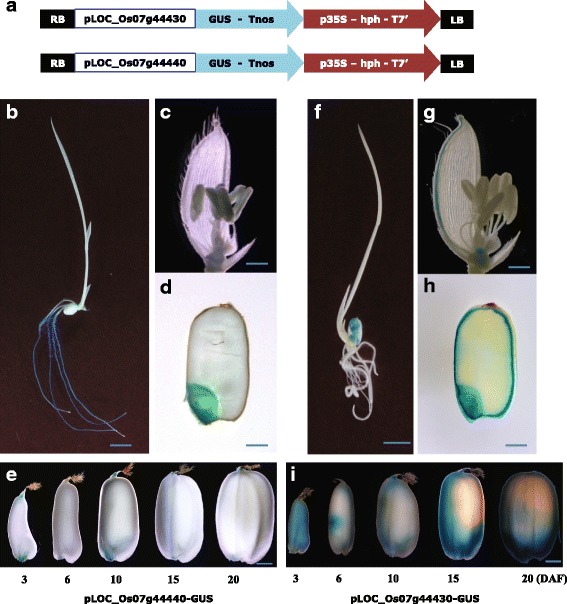
Validation of differential expression patterns for two tandemly duplicated rice genes using *GUS* reporter system. **a** Maps of promoter–*GUS* vector for tandemly duplicated genes LOC_Os07g44430 (*upper*) and LOC_Os07g44440 (*lower*). **b-e** *GUS* activity in transgenic plant carrying pLOC_Os07g44440–*GUS* vector, using samples from whole seedling (**b**), flower **(c)**, germinating seed **(d)**, and developing seed at 3, 6, 10, 15, and 20 DAP. (**e**). **f-i** *GUS* activity in transgenic plant carrying pLOC_Os07g44430–*GUS* vector, using samples from whole seedling (**f**), flower (**g**), germinating seed (**h**), and developing seed at 3, 6, 10, 15, and 20 DAP (**i**)

Expression patterns differed between the two tandemly duplicated rice PRX genes in the 1-Cys Prx subgroup. For closer examination, we constructed vectors expressing GUS under the control of the promoters of these two genes. Vector maps are presented in Fig. 3A and detailed information for the promoter analyses is provided in Additional file 3: Table S1. For the assays, we used whole seedlings, leaves, root cross sections, flowers, developing seeds, and cross sections of germinating seeds. As expected from the results of meta-expression analysis in those six tissues/organs, we confirmed root-preferential expression for *Os1-CysPrxB* and endosperm-preferential expression for *Os1-CysPrxA* (Fig. 3). We also found, unpredictably, that *Os1-CysPrxB* showed an embryo-preferential pattern in both developing and germinating seeds. This should have been expected of a root-preferential gene because developing rice seeds form precursors of embryonic roots at 10 or 12 DAP (Itoh et al. 2005). All of these data suggested that these closely linked genes have specialized roles in the development of two major seed organs. *Os1-CysPrxA* was expressed in the aleurone layer of the endosperm in developing and germinating seeds. Expression of this gene expanded from the aleurone layer to the central region of the endosperm during development. Therefore, we hypothesized that these two genes contain CREs in their promoter regions that are essential for differential expression.

### Analysis of CREs responsible for tissue- or organ-preferential expression patterns of tandemly duplicated *Os1-CysPrxA* and *Os1-CysPrxB*

**Fig. 4:**
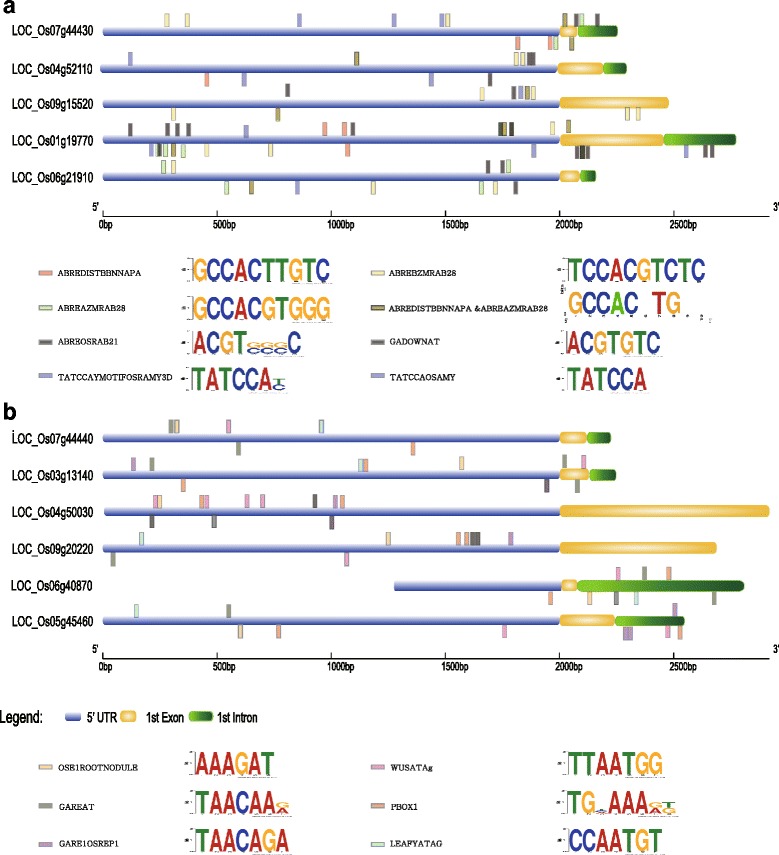
Identification of CREs responsible for unique expression patterns of two tandemly duplicated rice PRX genes. Mapping, names, and logos **a** of seven CREs to promoters of *LOC_Os07g44430* and its co-expressed genes. Mapping, names, and logos **b** of six CREs to promoters of *LOC_Os07g44440* and its co-expressed genes

To identify the unique CREs that determine root/embryo (*Os1-CysPrxB/LOC_Os07g44440*)- or endosperm (*Os1-CysPrxA/LOC_Os07g44430*)-preferential expression, we first found four more genes that showed expression patterns similar to *Os1-CysPrxA*, and five more with expression similar to *Os1-CysPrxB*. For this, we utilized top Pearson correlation coefficient (PCC) values obtained with the anatomy based-co-expression network tool installed in GENEVESTIGATOR (Hruz et al. 2008; Additional file 4: Figure S3). In silico analysis of CREs in promoter regions that included the first intron or exon (in the case of genes without an intron) of five genes in the *Os1-CysPrxA* group and six genes in the *Os1-CysPrxB* group revealed the presence of 40 CREs common to the promoter regions of both groups. This was accomplished using the PLANTPAN 2.0 database (Chow et al. 2016). The *Os1-CysPrxA* group contained 65 CREs in common, from which we selected 25 unique CREs in the promoters of that group as candidate CREs responsible for endosperm-preferential expression after 40 CREs were removed that are conserved in the promoters of the *Os1-CysPrxB* group (Additional files 5 and 6: Tables S2, S3). Of most interest to us were the four abscisic acid (ABA)-responsive CREs as well as those related to seed development, i.e., ABREDISTBBNNAPA/GCCACTTGTC, ABREAZMRAB28/GCCACGTGGG, ABREBZMRAB28/TCCACGTCTC, and ABREOSRAB21/ACGTSSSC. We also found three related to seed germination, i.e., GADOWNAT/ACGTGTC, TATCCAYMOTIFOSRAMY3D/TATCCAY, and TATCCAOSAMY/TATCCA, in the promoters of the Os1-CysPrxA group (Additional files 6 and 7: Tables S3, S4). Of the selected CREs for endosperm-preferential expression, TATCCAYMOTIFOSRAMY3D was more frequently identified than the others in those promoter regions (Fig. 4). The TATCCAYMOTIFOSRAMY3D motif was originally identified in the 5′ upstream regulatory region of rice α-*Amy3D* and is essential for the regulation of this gene by sugars (Lu et al. 1998; Lu et al. 2002). The TATCCAOSAMY motif (TATCCA) is part of TATCCAYMOTIFOSRAMY3D and is a binding site of OsMYBS2 and OsMYBS3, which function in sugar- and hormone-mediated regulation of alpha-amylase gene expression (Lu et al. 1998; Lu et al. 2002). The alpha-amylase in the aleurone layer plays an important role in hydrolyzing the endosperm starch into sugars that can be metabolized during seed germination, thereby providing energy for shoot growth (Murai et al. 1985; Mitsui et al. 1985). Whereas ABREDISTBBNNAPA is required for seed-specific expression and ABA-responsiveness (Ezcurra et al. 2000; Busk and Pages 1997, 1998), the other ABA-responsive CREs -- ABREAZMRAB28 (GCCACGTGGG) and ABREBZMRAB28 (TCCACGTCTC) -- are ABA-responsive element A (ABRE A) and ABA-responsive element B (ABRE B), which occur in the promoter of *Rab28* from *Zea mays* (maize) (Busk and Pages 1997, 1998). In addition, *Rab28* is highly expressed during the late stage of embryo development and is stimulated in response to ABA (Niogret et al. 1996; Busk et al. 1999). A role for ABRE A in the endosperm of developing or germinating seeds is easily estimated because ABA-mediating drought response is closely associated with seed ripening process. These ABA-responsive CREs might be involved in reducing the water content during the process of seed maturation.

Within the *Os1-CysPrxB* group, we identified 50 CREs common to their promoters (Fig. 4; Additional files 3 and 7: Tables S1, S4). Among these, 10, including PBOX1 (TGRAAG), GAREAT (TAACAAR), GARE1OSREP1 (TAACAGA), OSE1ROOTNODULE (AAAGAT), LEAFYATAG (CCAATGT), and WUSATAg (TTAATGG) were unique to that group and did not occur in the *Os1-CysPrxA* group (Additional files 8 and 9: Tables S5, S6). GAREAT and GARE1OSREP1 are involved in gibberellin (GA)-responsiveness, suggesting that they help regulate the GA signaling pathway in rice embryos (Sutoh and Yamauchi 2003; Ogawa et al. 2003). Furthermore, OSE1ROOTNODULE (AAAGAT) is an organ-specific element (OSE) for the regulation of an arbuscular mycorrhizal and nodule-inducing leghaemoglobin gene in root nodules (Stougaard et al. 1990; Vieweg et al. 2004; Fehlberg et al. 2005); and LEAFYATAG and WUSATAg are present in the promoter of a rice WUSCHEL-type homeobox gene that is expressed in the central cells of quiescent centers in the root apical meristem (Kamiya et al. 2003). Both GAREAT and GARE1OSREP1 might be expressed preferentially in the embryo while LEAFYATAG, WUSATAg, and OSE1ROOTNODULE show root-preferential expression as members of the *Os1-CysPrxB* group. The other CREs not mentioned here might have novel roles in driving root/embryo- or endosperm-preferential expression. Future experiments will be necessary to confirm our predictions (Additional files 5 and 8: Tables S2, S5).

### Two PRXs showing leaf- or shoot-preferential expression patterns are involved in the *DXR*-mediated light-response pathway to remove ROS during photosynthesis

**Fig. 5:**
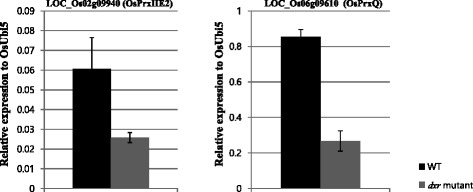
RT-PCR analysis of *OsPrxQ/LOC_Os06g09610* and *OsPrxIIE2/LOC_Os02g09940* in *dxr* mutant. Expression level relative to *OsUbi5* was compared between WT segregant and *dxr* mutant

Analysis with various tissues/organs revealed three *PRX*s that are most highly expressed in the leaf and shoot. Recently, we used a T-DNA insertion to identify a rice *1-deoxy-D-xylulose 5-phosphate reductoisomerase* (*dxr*) mutant with an albino phenotype. That finding indicated that the defective gene is involved in the light-response pathway (Jung et al. 2008). The DXR enzyme is active in the second step of the MEP pathway for isoprenoid biosynthesis, which interconverts 1-deoxy-D-xylulose 5-phosphate and MEP. Of the three rice *PRX*s (*OsPrxIIE2, Os2-CysPrxA*, and *OsPrxQ*) that show leaf/shoot-preferential expression, both *OsPrxIIE2* and *OsPrxQ* were significantly down-regulated in the *dxr* mutant when compared with the wild type (WT). This was demonstrated in our previous analyses via quantitative RT-PCR and microarray data (Chandran et al. 2016c), and it further supports our conclusion that these rice PRX genes are involved in the *DXR*-mediated light-response pathway (Fig. 5, Additional file 10: Figure S4).

### Regulatory network mediated by rice PRX genes

**Fig. 6.**
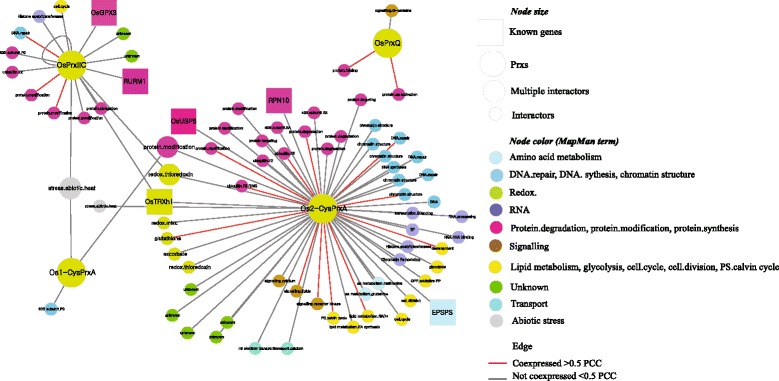
Working model of rice PRXs for regulatory pathway using refined protein–protein interaction network. Predictions were made with RIV, which revealed interactions with 161 proteins mediated by four PRX proteins. *Red edges* (*lines*) indicate elements with PCC values >0.5; *large rectangles* in node, three interaction proteins with known functions identified from OGRO database; *large circles*, PRX family members; *medium circles*, interactors showing multiple interactions with PRX proteins. MapMan annotation is indicated by different node colors: *bright green*, redox class proteins, including peroxiredoxins and thioredoxins; *pale blue*, proteins involved in amino acid metabolism; *yellow*, proteins involved in cell cycle, lipid metabolism, photosynthesis, and development; *blue*, proteins involved in DNA synthesis and repair; *pink*, proteins involved in protein degradation and post-translational modification; *purple*, proteins involved with RNA, mostly transcriptional regulators; *brown*, signaling proteins; *gray*, abiotic stress proteins; *light blue*, transporter class proteins; *green*, proteins with unknown functions (see Additional file 11: Table S7).

As part of our exploration of the regulatory mechanism mediated by rice PRX genes, we recently performed a protein–protein interaction network analysis in the Rice Interaction Viewer (RIV; http://bar.utoronto.ca/interactions/cgi-bin/rice_interactions_viewer.cgi) (Chandran et al. 2016a). Among the 10 rice PRXs, four (Os1-CysPrxA, Os2-CysPrxA, OsPrxQ, and OsPrxIIC) encode 82 predicted interacting proteins (Fig. 6): Os2-CysPrxA has 60; OsPrxIIC, 16; Os1-CysPrx A, 3; and OsPrxQ, 3 (Fig. 6).

To develop a more detailed protein–protein interaction network, we incorporated the MapMan classification data (indicated by node color) in Additional file 11: Table S7. This regulatory network provides insight into the molecular functions of the rice PRXs (Fig. 6). We also integrated PCC values based on meta-expression data for various tissues/organs in the network. In all, 12 genes had PCC values >0.5 for *Os2-CysPrxA*, the PRX gene most highly expressed in leaves and shoots; three genes had PCC values >0.5 for the ubiquitously expressed *OsPrxIIC*; and two genes had PCC values >0.5 for *OsPrxQ*, with a leaf-preferential expression pattern. We did not identify co-expressed genes for *Os1-CysPrxA*, which had a seed-preferential expression pattern (Fig. 6).

Functions were characterized for three genes in this network: *LOC_Os07g28280*, encoding a rice ubiquitin-related modifier 1 (RURM1) involved in mobilizing Active MITE mPing (Tsukiyama et al. 2013); *LOC_Os03g13970/RPN10*, encoding the 26S proteasome non-ATPase regulatory subunit 4, involved in canavanine resistance (Takase et al. 2004); and *LOC_Os06g04280*, encoding 5-enolpyruvoylshikimate-3-phosphate synthase (EPSPS), which is involved in the production of tillers, panicles, and seeds (Wang et al. 2014). These known genes linked to PRXs can now provide valuable clues about the functions of the unknown PRXs. Elements with multiple interactions in the network will also enable greater refinement of predicted protein–protein interaction networks when compared with other interactors. In fact, we identified four such elements in our network: *LOC_Os08g39140*, encoding heat shock protein 90 (HSP90) that interacts with OsPrxIIC and Os1-CysPrxA; *LOC_Os07g44710*, encoding a calcium-mediating protein kinase (CAMK); and *LOC_Os07g08840* and *LOC_Os03g58630*, which encode thioredoxins that interact with Os1-CysPrxA and Os2-CysPrxA. Among these interacting proteins in the primary network, we considered those showing co-expression or multiple interactions with PRX proteins to be more reliable candidates for constructing a regulatory network associated with PRXs in rice. Such a refined and integrated network will be a useful molecular frame for designing further studies.

## Discussion

### What is the functional similarity or diversity between rice and *Arabidopsis* PRXs?

Peroxiredoxins are universal among bacteria, fungi, animals, cyanobacteria, and plants (Dietz 2003). Among all of these organisms, PRXs are most abundant in plants, most likely because they must process large quantities of ROS that are produced during photosynthesis. We found that OsPrxIIC, a Type-II Prx, clustered with AtPrxIIA, AtPrxIIB, AtPrxIIC, and AtPrxIID, while OsPrxIIE1, OsPrxIIE2, and OsPrxIIE3 clustered with AtPrxII E (Fig. 1). Therefore, those three members of the Type-II subgroup mean that further expansion was initiated from AtPrxIID in *Arabidopsis* and OsPrxIIE3 in rice. Because those subgroup members show a ubiquitous expression pattern, we might infer that these genes primarily have housekeeping roles. For the newly expanded members, their tissue-preferred roles are to be expected. For example, AtPrxIIC in *Arabidopsis* might function in the processing of ROS produced from roots and pollen, while OsPrxIIE2 in rice might be active in processing ROS produced from leaves and shoots. Genes in the *1-Cys Prx* subgroup in those two species were preferentially expressed in endosperm, based on their meta-expression patterns. However, the rice members of that subgroup might have unique roles, based on our finding that, unlike its fellow members, *Os1-CysPrxB* was preferentially expressed in the root and embryo. Although *AtPrxQ* and *OsPrxQ* have similar patterns, being highly expressed in leaves and shoots, the former showed greater expression in flowers and seeds than did the latter. All genes in the 2-CysPrx subgroup, except for *Os2-CysPrxB*, which had very low expression in most tested tissues/organs, were commonly expressed at high levels in leaves and shoots. However, expression in the flowers and seeds was higher for *Arabidopsis* members than for rice members in that subgroup. Rice genes in the *2-CysPrx* and *PrxQ* subgroups were more likely to function in leaves and shoots when compared with the *Arabidopsis* members, indicating that rice requires them for survival when growing under higher light intensities than are associated with *Arabidopsis*. All of these results suggest that functional diversity of some PRXs has developed evolutionarily between the two species.

### A hypothetical model for the rice PRX-mediated pathway for ROS processing

Our predicted protein–protein interaction network analysis presented 82 interactors associated with four rice PRXs that showed ubiquitous or leaf/shoot-preferential expression patterns. Two of them already have known functions, including Os2-CysPrxA, which interacts with h-type thioredoxin 1 (OsTRXh1) and is involved in ABA sensitivity during seed germination and the early seedling stage (Zhang et al. 2011). This PRX also interacts with EPSPS, which participates in tolerance to drought and salinity (Tuteja et al. 2013); RPN10, which is related to canavanine resistance as a subunit of 26S proteasome (Takase et al. 2004), and the ubiquitin specific protease 6 (OsUBP6), which is related to growth rates during the seedling stage as well as the transport of iron into the mitochondria (Moon et al. 2009). These linkages suggest potential roles for Os2-CysPrxA in regulating ABA sensitivity, abiotic stress tolerance, normal plant development, and ubiquitin-dependent proteolysis at the cellular and molecular levels. Its higher expression in leaves than in other tissues suggests that its primary role is in leaf development, as mediated by the cellular and molecular functions mentioned above.

The second PRX with a known function is OsPrxIIC, which interacts with glutathione peroxidase 3 (OsGPX3) for hydrogen peroxide homeostasis; RURM1, involved in the mobilization of Active MITE mPing as a ubiquitin-related protein; and the previously discussed OsTRXh1. Based on its pattern of ubiquitous expression, OsPrxIIC may have a housekeeping function through its association with ROS-processing enzymes (including glutathione peroxidase and thioredoxin) or else in ubiquitin-related processes.

The signaling cascades of PRXs that occur in response to stresses have been analyzed for 1-CysPrx and 2-CysPrx in *Arabidopsis* (Dietz 2016). A redox-dependent retrograde signaling pathway from the chloroplast to the nucleus has been reported for 2-CysPrx (Baier and Dietz 2005). Because of its leaf/shoot-preferential expression pattern, Os2-CysPrxA might be a functional ortholog of *Arabidopsis* 2-CysPrx, thereby suggesting that rice has a potential ortholog for its redox-dependent retrograde signaling pathway. Here, we identified two candidate PRXs for the light-dependent signaling pathway to process ROS. Both *OsPrxIIE2* and *OsPrxQ* were down-regulated in the *dxr* mutant, which is defective in the light response. These PRXs might have separate roles in different retrograde signaling pathways or might combine with the signaling pathway of Os2-CysPrxA. This possibility remains to be elucidated through further studies.

### Tandemly duplicated PRXs with differential expression patterns increase functional diversity in rice

In *Arabidopsis*, *AtPrxIIC, AtPrxIIB*, and *AtPrxIIA* are tandem duplicates that show different expression patterns. In particular, *AtPrxIIC* is expressed at a high level in pollen but at lower levels in flowers and seed, while the opposite is true for *AtPrxIIB*. This suggests mutually antagonistic regulation. In addition, expression of *AtPrxIIA* is very low overall, possibly because of suppression via epigenetic regulation. The tandemly duplicated PRX genes in rice, *Os1-CysPrxA* and *Os1-CysPrxB*, also have dissimilar expression patterns. A Blast2 alignment of these PRXs indicated 89% amino acid sequence similarity (Additional file 12: Figure S5), which might mean that they are functionally redundant. However, their meta-expression patterns differed among tissues/organs. Whereas *Os1-CysPrxB* showed a root/embryo-preferential expression pattern, *Os1-CysPrxA* was preferentially expressed in the endosperm. This indicated that their promoters contain unique *cis*-acting elements. In our study, we focused on six CREs unique to the promoters of genes present in the *Os1-CysPrxB* subgroup but not in the *Os1-CysPrxA* subgroup. Three of the six -- LEAFYATAG, WUSATAg, and OSE1ROOTNODULE –appear necessary for root-preferential expression while two others -- GAREAT and GARE1OSREP1 – are related to embryo-preferential expression. For those expressed preferentially in the endosperm and belonging to the *Os1-CysPrxA* subgroup, we focused on seven CREs. Of them, ABREDISTBBNNAPA, ABREAZMRAB28, ABREBZMRAB28, and ABREOSRAB21 might be required for the ABA response during seed maturation while GADOWNAT, TATCCAYMOTIFOSRAMY3D, and TATCCAOSAMY might be needed for the processing of endosperm after germination. However, further investigations are necessary to evaluate the functionality of these unique CREs suggested here.

Studies of functional genomics that utilize gene-indexed mutants or genome-editing methods will provide more insight into the role of these rice PRXs. In addition, functional classifications retrieved from MapMan annotations of the interactors linked with four rice PRXs might help researchers elucidated the yet-unknown molecular mechanisms by which PRXs mediate stress responses.

## Conclusions

We carried out comparative expression analysis of rice and Arabidopsis PRX family genes which suggests conserved or diversified roles between the two species, leading the identification of tandemly duplicated rice PRX genes in the 1-CysPrx subgroup, Os1-CysPrxA and Os1-CysPrxB, differentially expressed in seeds. Os1-CysPrxB showed embryo- or root-preferential expression, while Os1-CysPrxA showed endosperm-preferential expression. Analysis of the cis-acting regulatory elements (CREs) revealed unique CREs responsible for embryo and root or endospermpreferential expression. In addition, the presence of leaf/shoot-preferential PRXs in rice suggests their evolutional requirement to survive in the growth environment with a higher light intensity when compared with that of Arabidopsis. Downregulation of two PRXs in the dxr mutant causing an albino phenotype implies that those genes have roles in processing ROS produced during photosynthesis. Predicted protein-protein network associated with four PRXs suggests useful regulatory model for further study.

## Methods

### Multiple sequence alignment and phylogenetic analysis

To perform a phylogenomic analysis of PRXs in rice and *Arabidopsis*, we collected 11 family members from the Rice Genome Annotation Project using locus IDs (http://rice.plantbiology.msu.edu/), and 10 *Arabidopsis* family members previously reported (Umate 2010). Multiple alignment of amino acid sequences was conducted with the ClustalX program, version 2.0.11 (Higgins et al. 1996). The phylogenetic analysis included MEGA 5.2 and the following parameters: Neighbor-Joining tree method, complete deletion, and bootstrap with 500 replicates (Tamura et al. 2011). The resulting phylogenic tree comprised 11 rice and 10 *Arabidopsis* PRX proteins (Umate 2010). Those from rice were classified into four subgroups based on a comparative analysis between rice and *Arabidopsis* (Fig. 1). We also generated a phylogenic tree for rice PRXs to integrate meta-expression data for six tissues/organs (Additional file 2: Figure S2).

### Meta-analysis of tissue-specific expression profiles

Integration of spatio-temporal expression profiles into a phylogenetic context can direct experimental strategies for further functional analysis (Jung et al. 2010). Therefore, we used meta-analysis of 17 tissue/organ-type expression profiles based on data from 983 Affymetrix arrays downloaded from the NCBI gene expression omnibus (http://www.ncbi.nlm.nih.gov/geo/) (Cao et al. 2012). We then uploaded the log_2_-normalized intensity data in a tab-delimited text format into the Multi Experiment Viewer (http://mev.tm4.org/#/welcome) and illustrated these data using heat maps (Additional file 2: Figure S2). In addition, we analyzed the meta-expression patterns of *Arabidopsis* PRX genes in six tissues/organs using the *Arabidopsis* Affymetrix microarray data series GSE5630, GSE5633, GSE5631, GSE5632, GSE5634, GSM943445, and GSM943446. Similar to the rice data analysis, we generated meta-expression data. To compare gene expression, we examined the data from six tissue/organ types from both rice and *Arabidopsis* (Fig. 1). This resulted in meta-expression data for all PRX genes, except for AtPrxIID, which is not present in the *Arabidopsis* Affymetrix array platform (GPL2025). The log_2_ intensities of microarray data differed between species, with the rice heat map ranging from 5 to 15 while the *Arabidopsis* heat map had a range from 5 to 13. Integrated meta-expression data were used to determine functional conservancy in terms of anatomy between rice and *Arabidopsis* PRX ortholog pairs.

### Predicted protein–protein interactions and PCC analysis

To infer the regulatory network of PRXs in rice, we conducted a predicted protein–protein interaction analysis in the RIV (Chandran and Jung 2014). After copying and pasting the locus IDs of the 10 PRX rice proteins into the RIV query box, we were able to identify interactions with 161 proteins that were mediated by four of those PRX proteins (Fig. 6). The interaction information presented in Additional file 11: Table S7 was then uploaded to Cytoscape (Shannon et al. 2003). The large circles (nodes) indicated PRXs and elements with known functions acquired from the Overview of Functionally Characterized Genes in Rice Online database (OGRO) database (Yamamoto et al. 2012), while the small circles indicated proteins predicted to interact with PRXs in rice. We then performed a PCC analysis between targets and interactors in RIV, using affymetrix expression data (Cao et al. 2012). This enabled us to identify elements with PCC values >0.5. In addition, three interaction proteins with known functions from the OGRO database were identified. The MapMan annotation indicated redox class proteins (including peroxiredoxins and thioredoxins), as well as proteins involved in amino acid metabolism, the cell cycle, lipid metabolism, photosynthesis, normal plant development, DNA synthesis and repair, protein degradation and post-translational modification, RNA (mostly transcriptional regulators), signaling proteins, abiotic stress proteins, and transporter class proteins. The annotations also indicated proteins with yet-unknown functions (Additional file 13: Table S8). The refined network with integrating omics data was utilized to infer regulatory pathways (Fig. 6).

### Plant materials

After sterilization with 50% (*w*/*v*) commercial bleach for 30 min with gentle shaking, rice seeds were germinated on a Murashige Skoog medium under controlled conditions (28 °C/25 °C day/night, 8-h photoperiod, and 78% relative humidity). In addition, we harvested flowers and pollen at the heading stage, and collected seeds at 10 and 15 DAP in greenhouse condition to extract total RNAs. All samples were directly frozen in liquid nitrogen and stored at −70 °C. For analyzing differential expression between the *dxr* mutant and the WT, we extracted total RNA from 7-day-old seedlings of both genotypes grown under greenhouse conditions.

### Promoter analysis using the *GUS* reporter gene

The *Os1-CysPrxA* promoter region (−1 to −2076 bp from the initiation ATG codon) and the *Os1-CysPrxB* promoter region (−1 to −1758 bp) were amplified with the 5-GTACCAGTTCGCCTCTAGAATTGAG-3 (Xba I) / 5-TCTCGAGGCGACGAACGACTGTGCTGC-3 (Xho I) primer set and the 5-ATCTAGAGTGGGTGTTGTGGTTGG-3 (Xba I) / 5- ACTCGAGATGAGGAATCGAGGATTAACC-3 (Xho I) primer set, respectively. The promoter DNA fragment was placed upstream of the *beta-glucuronidase* (*GUS*) reporter gene located in binary vector pGA3383 (Kim et al. 2009). Transgenic plants harboring the above construct were obtained in the *japonica* cultivar ‘Dongjin’ background through the *Agrobacterium tumefaciens* method of transformation (Lee et al. 1999).

To monitor *GUS* expression, we used 7-day-old whole seedlings, plus mature leaves, roots, flowers, developing seeds, and germinating seeds for two transgenic lines and performed GUS staining as described by Jefferson et al. (1987). As we have recently described (Hong et al., 2017) photographs of the GUS-assayed seedlings, mature leaves, flowers, developing seeds, and germinating seeds were produced using an EOS 560 digital camera (Canon, Tokyo, Japan) while those of hand-sectioned roots and germinating seeds were obtained using a BX61 microscope (Olympus, Tokyo, Japan).

### Analysis of *cis*-regulatory elements

To compare the CREs responsible for differences in promoter-mediated spatial expression between *Os1CysPrxA* and *Os1-CysPrxB*, we first used the co-expression tool in GENEVESTIGATOR (Zimmermann et al. 2004) to identify four more genes showing similar expression patterns with *Os1-CysPrxA* and five for *Os1-CysPrxB*. We then extracted 2-kb sequences upstream of ATG for these 11 genes from PLANTPAN (http://plantpan2.itps.ncku.edu.tw) (Chow et al. 2016), and analyzed the CREs in promoters using PLACE (Higo et al. 1998). Those CREs were aligned with the Motif Alignment and Search Tool (Bailey et al. 2009; Fig. 4 and Additional file 3: Table S1). Known target motifs were selected based on *P*-values ≤0.05.

### RNA extraction, semi-quantitative RT-PCR, and real-time PCR

Roots and shoots from rice seedlings were frozen in liquid nitrogen and ground with a Tissue Lyser II (Qiagen, Hilden, Germany). Their RNAs were extracted with the RNAiso Plus Kit according to the manufacturer’s protocol (Takara Bio, Kyoto, Japan). To determine tissue-specific expression patterns by RT-PCR, we used a primer set for *rice ubiquitin 5* (*OsUbi5*, *LOC_Os01g22490*) (Jain et al. 2006). The PCR conditions included 22 to 38 cycles of 95 °C for 30 s, 57 °C for 30 s, and 72 °C for 1 min 30 s. For real-time PCR, the cycling conditions were 95 °C for 15 s, 57 °C for 30 s, and 72 °C for 60 s. This experiment was repeated three times using the same control primer sets. Relative transcript levels and fold-changes were calculated by the 2^-∆Ct^ and 2^-∆∆Ct^ methods (Schmittgen and Livak 2008), respectively.

To compare the expression of PRX genes between the *dxr* mutant and WT plants, we first balanced the transcripts between their leaves using *OsUbi5* as we have previously described (Chandran et al. 2016b)*.* We then conducted an RT-PCR analysis with leaf samples to compare band intensities between the two genotypes. All primers used for RT-PCR analysis and vector construction are described in Additional file 14: Table S9.

## Additional files

Additional file 1: Figure S1. Maps showing chromosomal localization of 10 rice (A) and 10 *Arabidopsis* (B) PRX genes. (JPEG 1224 kb)

Additional file 2: Figure S2. Detailed meta-expression analysis using 995 tissues/organs of rice PRX family genes. (JPEG 1235 kb)

Additional file 3: Table S1. Summary of selected promoter regions and PCC values for individual members in *Os1-CysPrxA* and *Os1-CysPrxB* groups. (ODS 16 kb)

Additional file 4: Figure S3. Meta-expression data for genes co-expressed with *Os1-CysPrxA* and *Os1-CysPrxB*, using anatomy tool in GENEVESTIGATOR. (JPEG 1461 kb)

Additional file 5: Table S2. Summary of 25 CREs uniquely identified in *Os1-CysPrxA* group compared with *Os1-CysPrxB* group*. (ODS 16 kb)*

Additional file 6: Table S3. Detailed promoter regions of 25 CREs uniquely identified in *Os1-CysPrxA* group compared with *Os1-CysPrxB* group. (ODS 87 kb)

Additional file 7: Table S4. Summary of CREs described in Fig. 4. (ODS 13 kb)

Additional file 8: Table S5. Summary of 10 CREs uniquely identified in *Os1-CysPrxB* group compared with *Os1-CysPrxA* group*. (ODS 14 kb)*

Additional file 9: Table S6. Detailed promoter regions of 10 CREs uniquely identified in *Os1-CysPrxB* group compared with *Os1-CysPrxA* group. (ODS 87 kb)

Additional file 10: Figure S4. Phenotype comparison between *dxr* mutant and WT, RT-PCR analysis of *OsPrxQ* and *OsPrxIIE2* in mutant, and microarray data analysis of *dx*r mutant/WT using Agilent 44 K array. (JPEG 50 kb)

Additional file 11: Table S7. Detailed information about genes integrated in nodes of protein–protein interaction network. (ODS 15 kb)

Additional file 12: Figure S5. Blast2 alignment of amino acid sequences from *Os1-CysPrxA* and *Os1-CysPrxB*. (PDF 41 kb)

Additional file 13: Table S8. Information about predicted protein–protein interaction network depicted in Fig. 6. (ODS 15 kb)

Additional file 14: Table S9. Primer sequences used for RT-PCR analyses (Figs. 2 and 5). (ODS 16 kb)

## Abbreviations

CAMK: Calcium-mediating protein kinase
CREs: cis-acting regulatory elements
DXR: 1- deoxy-D-xylulose 5-phosphate reductoisomerase
EPSPS: 5-enolpyruvoylshikimate-3-phosphate synthase
GPX3: Glutathione peroxidase 3
HSP90: Heat shock protein 90
PRXs: Peroxiredoxins
ROS: Reactive oxygen species
RURM1: Rice ubiquitin-related modifier 1
TRXh1: h-type thioredoxin 1
UBP6: Ubiquitin specific protease 6

## References

[CR1] Baier M, Dietz KJ (1999). Protective function of chloroplast 2-cysteine peroxiredoxin in photosynthesis. Evidence from transgenic Arabidopsis. Plant Physiol.

[CR2] Baier M, Dietz KJ (2005). Chloroplasts as source and target of cellular redox regulation: a discussion on chloroplast redox signals in the context of plant physiology. J Exp Bot.

[CR3] Bailey TL, Boden M, Buske FA, Frith M, Grant CE, Clementi L, Ren J, Li WW, Noble WS (2009). MEME SUITE: tools for motif discovery and searching. Nucleic Acids Res.

[CR4] Bhatt I, Tripathi BN (2011). Plant peroxiredoxins: catalytic mechanisms, functional significance and future perspectives. Biotechnol Adv.

[CR5] Busk PK, Pages M (1997). Protein binding to the abscisic acid-responsive element is independent of VIVIPAROUS1 in vivo. Plant Cell.

[CR6] Busk PK, Pages M (1998). Regulation of abscisic acid-induced transcription. Plant Mol Biol.

[CR7] Busk PK, Pujal J, Jessop A, Lumbreras V, Pagès M (1999). Constitutive protein-DNA interactions on theabscisic acid-responsive element before and after developmental activation of the rab28 gene. Plant Mol Biol.

[CR8] Cao P, Jung KH, Choi D, Hwang D, Ronald PC (2012) The Rice Oligonucleotide Array database: an atlas of rice gene expression. Rice 5:17 10.1186/1939-8433-5-17 PMC4883718 24279809

[CR9] Chandran AKN, Jung KH (2014). Resources for systems biology in rice. J Plant Biol.

[CR10] Chandran AKN, Bhatnagar N, Kim B, Jung KH (2016). Genome-wide identification and analysis of rice genes to elucidate morphological agronomic traits. J Plant Biol.

[CR11] Chandran AKN, Jeong HY, Jung KH, Lee C (2016). Development of functional modules based on co-expression patterns for cell-wall biosynthesis related genes in rice. J Plant Biol.

[CR12] Chandran AKN, Lee GS, Yoo YH, Yoon UH, Ahn BO, Yun DW, Kim JH, Choi HK, An G, Kim TH, Jung KH (2016). Functional classification of rice flanking sequence tagged genes using MapMan terms and global understanding on metabolic and regulatory pathways affected by *dxr* mutant having defects in light response. Rice (N Y).

[CR13] Chow CN, Zheng HQ, Wu NY, Chien CH, Huang HD, Lee TY, Chiang-Hsieh YF, Hou PF, Yang TY, Chang WC (2016). PlantPAN 2.0: an update of plant promoter analysis navigator for reconstructing transcriptional regulatory networks in plants. Nucleic Acids Res.

[CR14] Dietz KJ (2003). Plant peroxiredoxins. Annu Rev Plant Biol.

[CR15] Dietz KJ (2011). Peroxiredoxins in plants and cyanobacteria. Antioxid Redox Signal.

[CR16] Dietz KJ (2016). Thiol-based Peroxidases and Ascorbate Peroxidases: why plants rely on multiple Peroxidase Systems in the Photosynthesizing Chloroplast?. Mol Cells.

[CR17] Dietz KJ, Jacob S, Oelze ML, Laxa M, Tognetti V, de Miranda SM, Baier M, Finkemeier I (2006). The function of peroxiredoxins in plant organelle redox metabolism. J Exp Bot.

[CR18] Ezcurra I, Wycliffe P, Nehlin L, Ellerstrom M, Rask L (2000). Transactivation of the *Brassica napus* napin promoter by ABI3 requires interaction of the conserved B2 and B3 domains of ABI3 with different cis-elements: B2 mediates activation through an ABRE, whereas B3 interacts with an RY/G-box. Plant J.

[CR19] Fehlberg V, Vieweg MF, Dohmann EM, Hohnjec N, Puhler A, Perlick AM, Kuster H (2005). The promoter of the leghaemoglobin gene VfLb29: functional analysis and identification of modules necessary for its activation in the infected cells of root nodules and in the arbuscule-containing cells of mycorrhizal roots. J Exp Bot.

[CR20] Haslekas C, Viken MK, Grini PE, Nygaard V, Nordgard SH, Meza TJ, Aalen RB (2003). Seed 1-cysteine peroxiredoxin antioxidants are not involved in dormancy, but contribute to inhibition of germination during stress. Plant Physiol.

[CR21] Higgins DG, Thompson JD, Gibson TJ (1996). Using CLUSTAL for multiple sequence alignments. Methods Enzymol.

[CR22] Higo K, Ugawa Y, Iwamoto M, Higo H (1998). PLACE: a database of plant Cis-acting regulatory DNA elements. Nucleic Acids Res.

[CR23] Hong HJ, Yoo HY, Park SA, Moon S, Kim SR, An G, Jung KH (2017) Genome-wide identification and extensive analysis of rice-endosperm preferred genes using reference expression database. J Plant Biol 60:249–258

[CR24] Hruz T, Laule O, Szabo G, Wessendorp F, Bleuler S, Oertle L, Widmayer P, Gruissem W, Zimmermann P (2008). Genevestigator v3: a reference expression database for the meta-analysis of transcriptomes. Adv Bioinforma.

[CR25] Itoh J, Nonomura K, Ikeda K, Yamaki S, Inukai Y, Yamagishi H, Kitano H, Nagato Y (2005). Rice plant development: from zygote to spikelet. Plant Cell Physiol.

[CR26] Jain M, Nijhawan A, Tyagi AK, Khurana JP (2006). Validation of housekeeping genes as internal control for studying gene expression in rice by quantitative real-time PCR. Biochem Biophys Res Commun.

[CR27] Jefferson RA, Kavanagh TA, Bevan MW (1987). GUS fusions: beta-glucuronidase as a sensitive and versatile gene fusion marker in higher plants. EMBO J.

[CR28] Jin GH, Gho HJ, Jung KH (2012). A systematic view of rice heat shock transcription factor family using phylogenomic analysis. J Plant Physiol.

[CR29] Jing LW, Chen SH, Guo XL, Zhang H, Zhao YX (2006). Overexpression of a chloroplast-located peroxiredoxin Q gene, SsPrxQ, increases the salt and low-temperature tolerance of Arabidopsis. J Integr Plant Biol.

[CR30] Jung KH, Lee J, Dardick C, Seo YS, Cao P, Canlas P, Phetsom J, Xu X, Ouyang S, An K, Cho YJ, Lee GC, Lee Y, An G, Ronald PC (2008). Identification and functional analysis of light-responsive unique genes and gene family members in rice. PLoS Genet.

[CR31] Jung KH, Cao P, Seo YS, Dardick C, Ronald PC (2010). The Rice Kinase Phylogenomics database: a guide for systematic analysis of the rice kinase super-family. Trends Plant Sci.

[CR32] Jung KH, Gho HJ, Nguyen MX, Kim SR, An G (2013). Genome-wide expression analysis of HSP70 family genes in rice and identification of a cytosolic HSP70 gene highly induced under heat stress. Funct Integr Genom.

[CR33] Kamiya N, Nagasaki H, Morikami A, Sato Y, Matsuoka M (2003). Isolation and characterization of a rice WUSCHEL-type homeobox gene that is specifically expressed in the central cells of a quiescent center in the root apical meristem. Plant J.

[CR34] Kim SR, Lee DY, Yang JI, Moon S, An G (2009). Cloning vectors for rice. J Plant Biol.

[CR35] Lee S, Jeon JS, Jung KH, An G (1999). Binary vectors for efficient transformation of rice. J Plant Biol.

[CR36] Lee KO, Jang HH, Jung BG, Chi YH, Lee JY, Choi YO, Lee JR, Lim CO, Cho MJ, Lee SY (2000). Rice 1Cys-peroxiredoxin over-expressed in transgenic tobacco does not maintain dormancy but enhances antioxidant activity. FEBS Lett.

[CR37] Lu CA, Lim EK, Yu SM (1998). Sugar response sequence in the promoter of a rice alpha-amylase gene serves as a transcriptional enhancer. J Biol Chem.

[CR38] Lu CA, Ho TH, Ho SL, Yu SM (2002). Three novel MYB proteins with one DNA binding repeat mediate sugar and hormone regulation of alpha-amylase gene expression. Plant Cell.

[CR39] Mitsui T, Akazawa T, Christeller JT, Tartakoff AM (1985). Biosynthesis of rice seed alpha-amylase: two pathways of amylase secretion by the scutellum. Arch Biochem Biophys.

[CR40] Moon YK, Hong JP, Cho YC, Yang SJ, An G, Kim WT (2009). Structure and expression of OsUBP6, an ubiquitin-specific protease 6 homolog in rice (*Oryza sativa* L.). Mol Cells.

[CR41] Murai H, Hara S, Ikenaka T, Goto A, Arai M, Murao S (1985). Amino acid sequence of protein alpha-amylase inhibitor from Streptomyces griseosporeus YM-25. J Biochem.

[CR42] Nguyen MX, Moon S, Jung KH (2013). Genome-wide expression analysis of rice aquaporin genes and development of a functional gene network mediated by aquaporin expression in roots. Planta.

[CR43] Nguyen VN, Moon S, Jung KH (2014). Genome-wide expression analysis of rice ABC transporter family across spatio-temporal samples and in response to abiotic stresses. J Plant Physiol.

[CR44] Nguyen QN, Lee YS, Cho LH, Jeong HJ, An G, Jung KH (2015). Genome-wide identification and analysis of *Catharanthus roseus* RLK1-like kinases in rice. Planta.

[CR45] Niogret MF, Culianez-Macia FA, Goday A, Mar Alba M, Pages M (1996). Expression and cellular localization of rab28 mRNA and Rab28 protein during maize embryogenesis. Plant J.

[CR46] Ogawa M, Hanada A, Yamauchi Y, Kuwahara A, Kamiya Y, Yamaguchi S (2003). Gibberellin biosynthesis and response during Arabidopsis seed germination. Plant Cell.

[CR47] Pospisil P (2009) Production of reactive oxygen species by photosystem II. BBA-bioenergetics 1787:1151–1160 10.1016/j.bbabio.2009.05.005 19463778

[CR48] Proteau PJ (2004). 1-Deoxy-D-xylulose 5-phosphate reductoisomerase: an overview. Bioorg Chem.

[CR49] Pulido P, Spínola MC, Kirchsteiger K, Guinea M, Pascual MB, Sahrawy M, Sandalio LM, Dietz KJ, González M, Cejudo FJ (2010). Functional analysis of the pathways for 2-Cys peroxiredoxin reduction in Arabidopsis thaliana chloroplasts. J Exp Bot.

[CR50] Romero-Puertas MC, Laxa M, Matte A, Zaninotto F, Finkemeier I, Jones AM, Perazzolli M, Vandelle E, Dietz KJ, Delledonne M (2007) S-nitrosylation of peroxiredoxin II E promotes peroxynitrite-mediated tyrosine nitration. Plant Cell 19:4120–4130 10.1105/tpc.107.055061 PMC2217656 18165327

[CR51] Schmittgen TD, Livak KJ (2008). Analyzing real-time PCR data by the comparative CT method. Nat Protoc.

[CR52] Shannon P, Markiel A, Ozier O, Baliga NS, Wang JT, Ramage D, Amin N, Schwikowski B, Ideker T (2003). Cytoscape: a software environment for integrated models of biomolecular interaction networks. Genome Res.

[CR53] Stougaard J, Jorgensen JE, Christensen T, Kuhle A, Marcker KA (1990). Interdependence and nodule specificity of cis-acting regulatory elements in the soybean leghemoglobin lbc3 and N23 gene promoters. Mol Gen Genet.

[CR54] Sutoh K, Yamauchi D (2003). Two cis-acting elements necessary and sufficient for gibberellin-upregulated proteinase expression in rice seeds. Plant J.

[CR55] Takase T, Yanagawa Y, Mitsuhara I, Ohashi Y, Hakagawa H, Hashimoto J (2004). Overexpression of a gene for 26S proteasome subunit RPN10 confers enhanced resistance to canavanine, an analog of arginine, in transgenic rice (*Oryza sativa* L.). Plant Biotechnol.

[CR56] Tamura K, Peterson D, Peterson N, Stecher G, Nei M, Kumar S (2011). MEGA5: molecular evolutionary genetics analysis using maximum likelihood, evolutionary distance, and maximum parsimony methods. Mol Biol Evol.

[CR57] Tsukiyama T, Teramoto S, Yasuda K, Horibata A, Mori N, Okumoto Y, Teraishi M, Saito H, Onishi A, Tamura K, Tanisaka T (2013). Loss-of-function of a ubiquitin-related modifier promotes the mobilization of the active MITE mPing. Mol Plant.

[CR58] Tuteja N, Sahoo RK, Garg B, Tuteja R (2013). OsSUV3 dual helicase functions in salinity stress tolerance by maintaining photosynthesis and antioxidant machinery in rice (*Oryza sativa* L. cv. IR64). Plant J.

[CR59] Umate P (2010). Genome-wide analysis of thioredoxin fold superfamily peroxiredoxins in Arabidopsis and rice. Plant Signal Behav.

[CR60] Vieweg MF, Fruhling M, Quandt HJ, Heim U, Baumlein H, Puhler A, Kuster H, Andreas MP (2004). The promoter of the *Vicia faba* L. leghemoglobin gene VfLb29 is specifically activated in the infected cells of root nodules and in the arbuscule-containing cells of mycorrhizal roots from different legume and nonlegume plants. Mol Plant-Microbe Interact.

[CR61] Wang W, Xia H, Yang X, Xu T, Si HJ, Cai XX, Wang F, Su J, Snow AA, Lu BR (2014). A novel 5-enolpyruvoylshikimate-3-phosphate (EPSP) synthase transgene for glyphosate resistance stimulates growth and fecundity in weedy rice (*Oryza sativa*) without herbicide. New Phytol.

[CR62] Yamamoto E, Yonemaru J, Yamamoto T, Yano M (2012). OGRO: the overview of functionally characterized genes in Rice online database. Rice.

[CR63] Zhang CJ, Zhao BC, Ge WN, Zhang YF, Song Y, Sun DY, Guo Y (2011). An Apoplastic H-type Thioredoxin is involved in the stress response through regulation of the Apoplastic reactive oxygen species in Rice. Plant Physiol.

[CR64] Zimmermann P, Hirsch-Hoffmann M, Hennig L, Gruissem W (2004). GENEVESTIGATOR. Arabidopsis microarray database and analysis toolbox. Plant Physiol.

