# Supplementary figures and images for: Comparative Expression Analysis of Rice and Arabidopsis Peroxiredoxin Genes Suggests Conserved or Diversified Roles Between the Two Species and Leads to the Identification of Tandemly Duplicated Rice Peroxiredoxin Genes Differentially Expressed in Seeds
Source: Rice (N Y). 2017 Jun 24;10:30. doi: 10.1186/s12284-017-0170-5 (PMC5483221; doi:10.1186/s12284-017-0170-5)

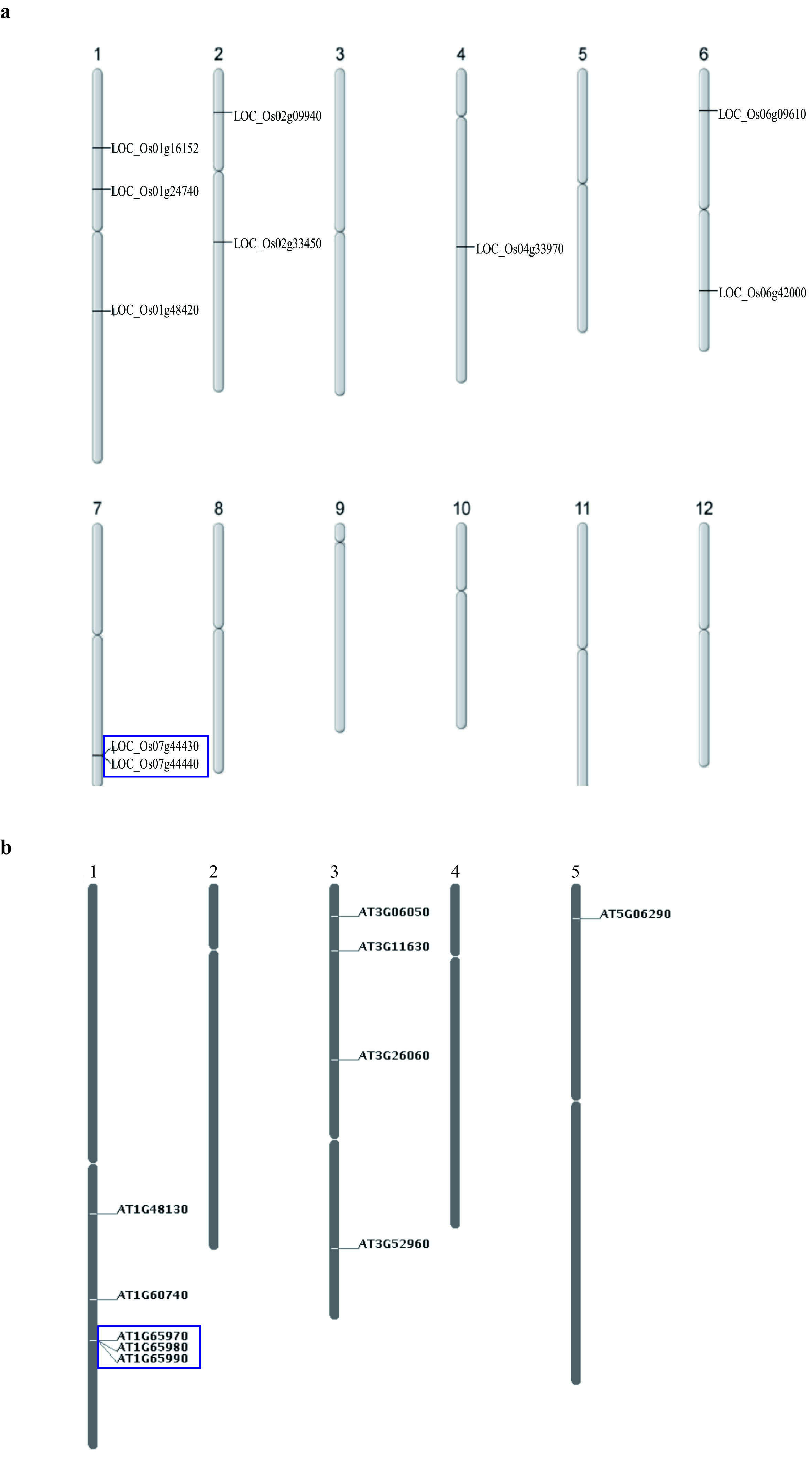

Supplement: Supplementary file 1 — Maps showing chromosomal localization of 10 rice (A) and 10 Arabidopsis (B) PRX genes. (JPEG 1224 kb) [file 12284_2017_170_MOESM1_ESM.jpg]

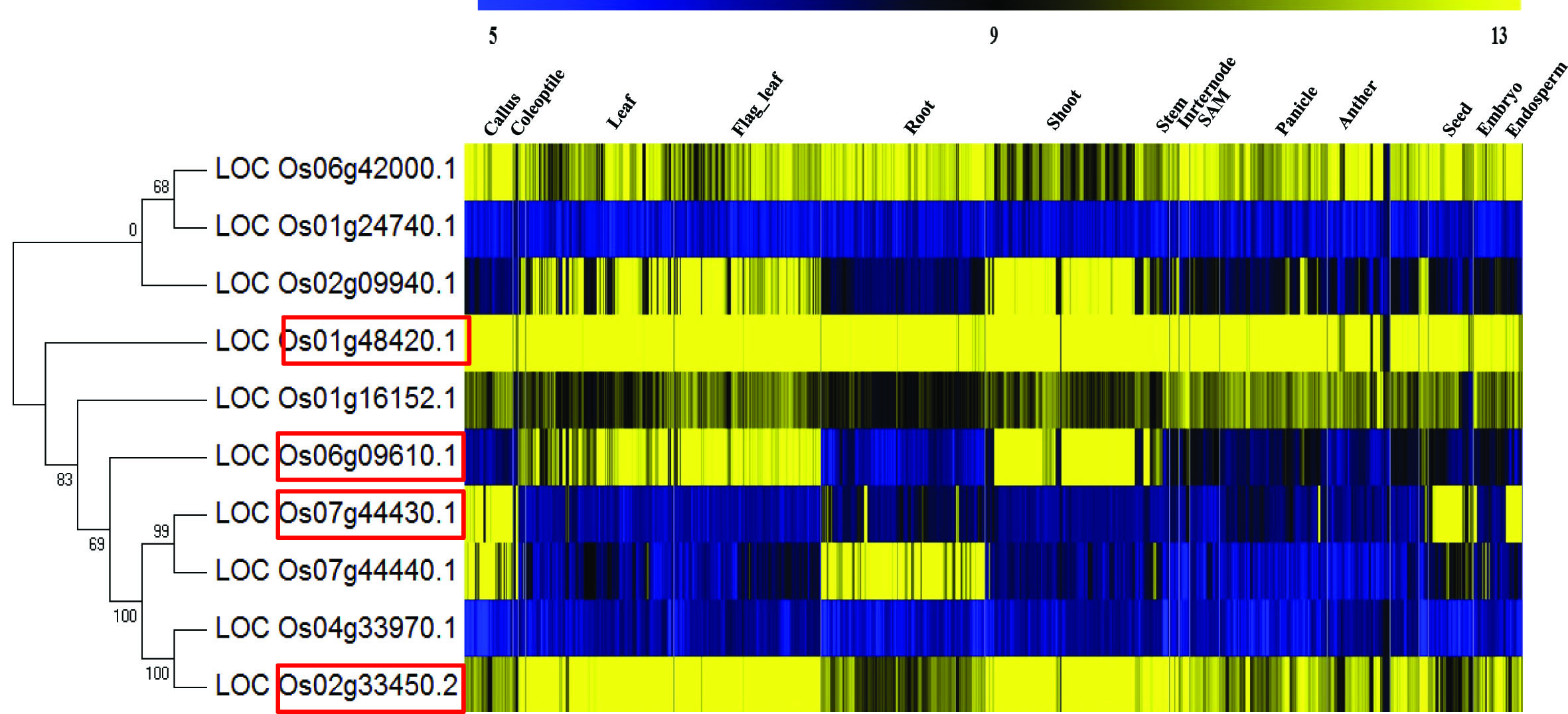

Supplement: Supplementary file 2 — Detailed meta-expression analysis using 995 tissues/organs of rice PRX family genes. (JPEG 1235 kb) [file 12284_2017_170_MOESM2_ESM.jpg]

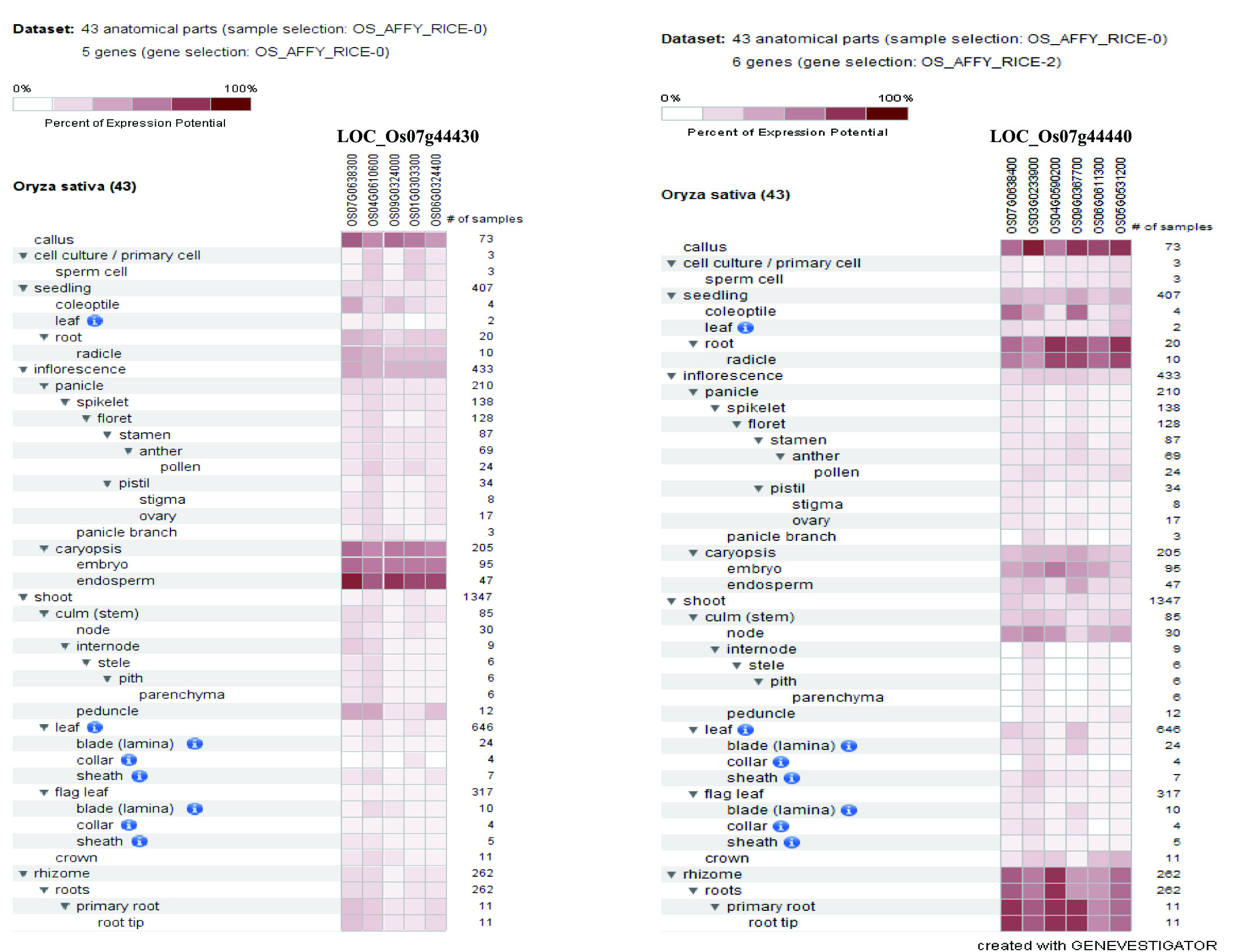

Supplement: Supplementary file 4 — Meta-expression data for genes co-expressed with Os1-CysPrxA and Os1-CysPrxB, using anatomy tool in GENEVESTIGATOR. (JPEG 1461 kb) [file 12284_2017_170_MOESM4_ESM.jpg]

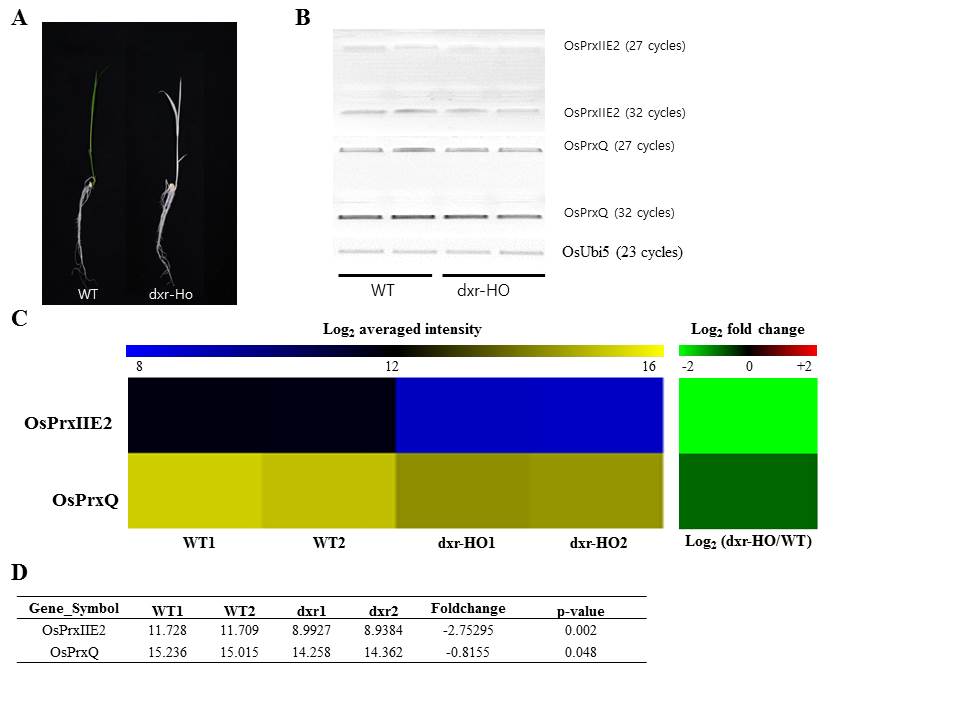

Supplement: Supplementary file 10 — Phenotype comparison between dxr mutant and WT, RT-PCR analysis of OsPrxQ and OsPrxIIE2 in mutant, and microarray data analysis of dxr mutant/WT using Agilent 44 K array. (JPEG 50 kb) [file 12284_2017_170_MOESM10_ESM.jpg]
