# Supplementary material for: Comparative Expression Analysis of Rice and Arabidopsis Peroxiredoxin Genes Suggests Conserved or Diversified Roles Between the Two Species and Leads to the Identification of Tandemly Duplicated Rice Peroxiredoxin Genes Differentially Expressed in Seeds
Source: Rice (N Y). 2017 Jun 24;10:30. doi: 10.1186/s12284-017-0170-5 (PMC5483221; doi:10.1186/s12284-017-0170-5)
Supplement: Supplementary file 12 — Blast2 alignment of amino acid sequences from Os1-CysPrxA and Os1-CysPrxB. (PDF 41 kb) [file 12284_2017_170_MOESM12_ESM.pdf]

Blast2 alignment of LOC_Os07g44430 and LOC_Os07g44440

Score	Expect	Method	Identities	Positives	Gaps	Frame
379 bits(973)	1e-138()	Compositional matrix adjust.	177/219(81%)	197/219(89%)	0/219(0%)	

Features:

```
LOC_Os07g44430 1 MPGLTLGDVVPDLELDTTHGKIRLHDFVGDAYVIIFSHPADFTPVCTTELSEMAGYAGEF  60
                 MPGLT+GD VP+LELD+THGKIR+HDFVGD YVI+FSHP DFTPVCTTEL+ MAGYA EF
LOC_Os07g44440 1 MPGLTIGDTVPNLELDSTHGKIRIHDFVGDTYVILFSHPGDFTPVCTTELAAMAGYAKEF  60

LOC_Os07g44430 61 DKRGVKLLGFSCDDVESHKDWIKDIEAYKPGRRVGFPIVADPDREAIRQLNMIDADEKDT  120
                  DKRGVKLLG SCDDV+SHKDWIKDIEAYKPG RV +PI+ADP REAI+QLNM+D DEKD+
LOC_Os07g44440 61 DKRGVKLLGISCDDVQSHKDWIKDIEAYKPGNRVTYPIMADPSREAIKQLNMVDPDEKDS  120

LOC_Os07g44430 121 AGGELPNRALHIVGPDKKVKLSFLFPACTGRNMAEVLRATDALLTAARHRVATPVNWKPG  180
                    GG LP+RALHIVGPDKKVKLSFL+PAC GRNM EV+RA DAL TAA+H VATPVNWKPG
LOC_Os07g44440 121 NGGHLPSRALHIVGPDKKVKLSFLYPACVGRNMDEVVRAVDALQTAAKHAVATPVNWKPG  180

LOC_Os07g44430 181 ERVVIPPGVSDEEAKARFPAGFETAQLPSNKCYLRFTQV  219
                   ERVVIPPGVSD+EAK +FP GF+TA LPS K YLRFT+V
LOC_Os07g44440 181 ERVVIPPGVSDDEAKEKFPQGFDTADLPSGKGYLRFTKV  219
```
